# Relationship between Depression and Strength Training in Survivors of the Ischemic Stroke

**DOI:** 10.2478/hukin-2014-0084

**Published:** 2014-11-12

**Authors:** Felipe José Aidar, Dihogo Gama de Matos, Ricardo Jacó de Oliveira, André Luiz Carneiro, Breno Guilherme de Araújo Tinôco Cabral, Paulo Moreira Silva Dantas, Victor Machado Reis

**Affiliations:** 1 Department of Sports Science, Exercise and Health of the Trás-os-Montes e Alto Douro University, Vila Real, Portugal.; 2 University of Brasília – Unb, Brasília, Distrito Federal, Brazil.; 3 Fire Brigade of Minas Gerais, 5th Battalion Fire Military Fire Brigade of the State of Minas Gerais, New Horizons Program, Uberlandia, Minas Gerais, Brazil.; 4 Department of Research in Sport, Health and Human Development - CIDESD, Vila Real, Portugal.; 5 State University at Montes Claros (UNIMONTES), Montes Claros, Brazil.; 6 Federal University of Rio Grande do Norte, Graduate Program in Physical Education – PPGEF, Rio Grande do Norte, Brazil.

**Keywords:** hypoxia-ischemia, brain, resistance training

## Abstract

The Cerebral Vascular Accident is responsible for a significant increase in the mortality rate in individuals who have suffered this condition, regardless of the level of subsequent disability. This study aimed to analyze the influence of a strength training program on indicators of depression in survivors of the ischemic stroke. The study sample included subjects from both genders who were divided into two groups: an experimental group (EG) consisting of 11 subjects aged 51.7 
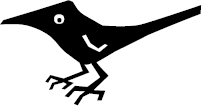
 8.0 years, and a control group (CG) consisting of 13 subjects aged 52.5 
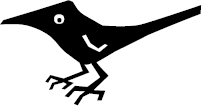
 7.7 years. The EG underwent 12 weeks of strength training. Assessment was made in the pre-test before training and at the re-test after 12 weeks of training. We used the Beck Depression Inventory and evaluated 1RM. Significant differences in depression were found between post-test and pretest measurements (Δ% = −21.47%, p = 0,021) in the EG; furthermore, there were significant differences in all indicators of depression between the EG and CG after completing 12 weeks of training. There were significant gains in strength of the EG in relation to the CG. There was a negative correlation between the strength gains as determined with the 1RM test and the levels of depression, especially in lower-limb exercises. The results of this study suggest that improvements in strength are negatively correlated with levels of depression. Improvements in strength are therefore associated with a reduction in levels of depression.

## Introduction

The Cerebral Vascular Accident (CVA) is responsible for a significant increase in the mortality rate in individuals who have suffered this condition, regardless of the level of subsequent disability (Aidar et al., 2011; Hankey, 2005; Sachdev et al., 2004; Rabelo and Néri, 2006). The odds this group of individuals will suffer from consequences that tend to be definitive are high (Devereux et al., 2010; Borghi-Silva et al., 2010; Fu et al., 2010). Among older adults, the sequelae of a stroke are considered the most common causes of disability, limiting daily physical tasks and thereby compromising the social activities that tend to alter the autonomy and impair psychosocial indicators (Aidar et al., 2011). In this sense, stroke survivors tend to have higher depressive symptoms than other individuals, resulting in reduced social interactions (Murrel et al., 2011). As a consequence, the deterioration of the psychological state results in reduced adherence to treatment (Yon Joo et al., 2010).

Physical exercise, particularly strength training, has been increasingly used in the prevention of new events and as a form of rehabilitation in stroke survivors (Patten et al., 2004; Hankey, 2006). The application of strength exercises has been pointed out as crucial not only for the improvement of daily activities, but has also been regarded as fundamental to improving posture and reducing pain (Haan et al., 1995; Beck et al., 1988; Gorenstein and Andrade, 1998). On the other hand, there are reports that strength training could increase spasticity in this population (Dunn et al., 1993). There is no consensus that strength training could be related to decreased risk of a stroke or decreased recurrence of a stroke (Tanaka-Matsumi and Kameoka, 1986), and there are few studies that relate the physical activities to psychosocial indicators, including depression, in stroke survivors.

The aim of this study was therefore to analyze the influence of strength training on depression in stroke survivors and individuals who showed sequelae due to a cerebrovascular event.

## Material and Methods

### Sample

The study began with 29 subjects, divided at random into two groups: a control group (CG; N-15, males: 52,3 ± 9,0 years; females: 50,8 ± 10,6 years) and an experimental group (EG; N=14, males: 52,8 ± 4,8 years; females: 52,6 ± 7,6 years). After the beginning of the intervention, early in the second week, two subjects left the EG program, so the EG was comprised of 12 subjects. During the remainder of the study there was one dropout from the EG during treatment and two dropouts from the CG for failing to attend the second time point, i.e., the post-testing. Thus, the EG was composed of 11 individuals (51.7 ± 8.0 years), six males and five females and the CG was composed of 13 individuals (52.5 ± 7.7 years), nine males and four females.

The intervention was conducted in the 3rd Battalion of the Military Fire Brigade of Belo Horizonte, Minas Gerais. The studies were carried out within the program New Horizons, a project developed by the aforementioned Fire Brigade which supports individuals with special needs at no charge. The criterion for inclusion in the “New Horizons” program was a “per-capita” income below the minimum wage in Brazil. Participation in the study was conditional on a medical authorization, with only clinically healthy subjects allowed to participate. Additional inclusion criteria for study participation were that the patient had suffered a stroke over a year prior to the study and at the time of the study be clinically stable, having hemiplegia or hemiparesis, and not having recurrent strokes. The sample was classified according to the Rankin scale (Haan et al., 1995). Thus, 18.2% of EG subjects had mild disability (mild), 63.6% had moderate disability, and 18.2% had higher disability. 15.4% of the subjects belonging to the CG had mild disability (mild), 61.5% had moderate disability and 23.1% had higher disability (Table 1). Asymptomatic subjects with a non-disabling deficit or with severe disabilities were excluded.

All volunteers were informed about the study design and signed a consent form in accordance with the Declaration of Helsinki (1964, revised in 1975, revised in 1983, revised in 1989, revised in 1996 and 2000). The procedures were approved by the 3rd Battalion of the Military Fire Brigade of Belo Horizonte, Minas Gerais ethics committee. All subjects underwent a pre-test, and the experimental group (EG) began the resistance exercises. The other group (CG) was not subjected to any physical activity until the end of the intervention.

### Instruments

#### Beck Depression Inventory

The Beck Depression Inventory (Beck Depression Inventory - BDI) (Beck et al., 1988; Gorenstein and Andrade, 1998) has been indicated as a valid means of self-assessment of depression in clinical practice in several countries (Gorenstein and Andrade, 1998). The original scale consists of 21 items, including symptoms and attitudes, whose ratings range from 0 to 3. The items refer to feelings of sadness, pessimism, sense of failure, lack of satisfaction, feelings of guilt, sense of punishment, self-deprecation, self-accusations, suicidal ideas, crying spells, irritability, social withdrawal, indecisiveness, distortion body image, work inhibition, sleep disturbance, fatigue, loss of appetite, weight loss, somatic preoccupation, decreased libido.

The Beck Inventory (Beck et al., 1988; Gorenstein and Andrade, 1998) allows various cutoffs, depending on the nature of the sample and study goals. For sampling with this test, the “Center for Cognitive Therapy” (Beck et al., 1988; Gorenstein and Andrade, 1998) recommends the following cutoff points: less than 10 = no or minimal depression, 10 to 18 = depression, mild to moderate; of 19 to 29 = depression, moderate to severe, 30 to 63 = severe depression.

#### Rating of perceived exertion (OMNI Scale)

The OMNI scale was used for the evaluation of perceived exertion (Gearhart et al., 2009). The instructions were carried out using pictures to describe the perception of effort during the familiarization and training session. The level of effort was related to a scale of Extremely Easy (0) to Extremely Hard (10), with the participant estimating the number corresponding to the intensity of exercise.

### Procedures

The subjects underwent four familiarization sessions before the pretest, three for the determination of a load and one to become familiar with the testing procedures. The activities were performed for 12 weeks, three times a week, with a minimum of 48 hours rest between each session. Each of these sessions had an average duration of 60 min and was conducted in the morning according to availability. The activities were composed of a warm up including a 10 – 15 min walk, followed by the following upper and lower body strength training exercises on Multipower equipment (Righetto, Brazil): bar guided squat, machine bench press, horizontal leg press, military press machine, abdominal crunch, front lat pull-downs and bar guided lunges (Fleck and Kraemer, 2004; Baechle and Groves, 2006; Hass et al., 2001). These subjects performed three sets of 8–10 repetitions with 2 min rest between sets (Aidar et al., 2007; Ouellette et al., 2004; Gearhart et al., 2009), observing the values of the OMNI Scale. The subjects were also instructed not to hold their breath during the exercises, thus avoiding the Valsalva maneuver and the associated increased blood pressure. Adjustments were made to ensure that all volunteers were able to perform all required activities during the study.

### Determination of Load

We performed a 1RM test, where each subject started the trials with a weight that they believed could be lifted only once using maximum effort. Weight was then added in increments until reaching the maximum load that could be lifted once. If the subject could not perform a single repetition, 2.4 to 2.5% of the load was removed and the trial was repeated (Aidar et al., 2012). The subjects rested 3–5 minutes between following attempts. All subjects underwent three test sessions of 1 RM for all exercises, with an interval of 48 to 72 hours between each session for evaluation of muscle strength. The test was preceded by a series of warm-ups (10 to 12 repetitions) with approximately 50% of the load to be used in the first attempt of a 1RM. The testing started two minutes after the warm up. It is noteworthy that the form and technique of each exercise was standardized and continuously monitored. In addition, the subjects performed the tests at the same time of day and did not perform other exercises during the experimental period (12 weeks).

The 1RM test was performed before the start of the intervention (pretest) and after 12 weeks (12 weeks) to determine the improvement in the 1RM test.

### Intensity Control

The scale of perceived exertion (OMNI) was used according to the procedure adopted by Lagally (2006) and Gearhart et al. (2009). The scale was presented to participants during the familiarization with strength training, which assigned a numerical value according to their overall perception of effort. The strength training load was adjusted during the intervention in relation to the recommended OMNI scale values of 6–8.

### Familiarization

After the individuals had filled in the questionnaires, two familiarization sessions were made with the exercises, as well as with the OMNI scale. During the familiarization, instructions were given on using the scale with the figures mentioned above, which would be used during the intervention. The scale was presented to participants during the strength training sessions, and they were requested to indicate a numerical value on the scale corresponding to their perceived exertion at that moment. The exercises were performed initially with one set, and in a second visit two sets, until the required number of sets (3) in the strength training protocol was reached.

### Statistics

We applied descriptive statistics and also checked the homogeneity of the sample through the Shapiro-Wilk test given the sample size. We used the Wilcoxon test between the pre and post-test for each group to check for changes in both groups at the end of 12 weeks. The analysis of the changes in depression and force levels was conducted using ANOVA (two way) with Bonferroni post hoc analysis. A correlation was made through the “r” according to Pearson normality to verify the correlation between the improvement in 1RM and the changes in the levels of depression over the 12 weeks. The significance level was set at p<0.05. The program used for data processing was the SPSS version 15.0.

## Results

There was a reduction (p < 0.05) in depression score in the EG, but not in the CG, over the 12-weeks (Table 2). In assessing the strength, there were improvements in all measures of strength in the EG that were not apparent in the CG (Table 3).

Correlation analysis revealed that there were negative correlations between all measures of strength gains as determined by the 1RM test, and levels of depression (p<0.05; Table 4).

## Discussion

The presented results show that the subjects in the pretest exhibited similar levels of depression, with depression being considered mild to moderate. The EG had scores averaging 17.7, slightly depressed at the pretest compared to 16.9 for the CG and very close to the cutoff point of 19. In the post-test, depression scores were 13.9 for the CG compared to 16.4 for the EG. In this sense, the exercise proved important in reducing depression, and therefore, the risk of mortality, in individuals affected by a stroke (Kim et al., 2001). To verify the benefits of physical activity in relation to psychosocial indicators in survivors of the ischemic stroke and sequelae due to the event, significant improvements were observed in subjects undergoing physical activity (strength training) (Sharp et al., 1997). Corroborating this effect, another study, related to strength training, showed an improvement in blood pressure in subjects who suffered a stroke and survived (Hann et al., 1995). Furthermore, improvements in force levels in stroke survivors submitted to strength training have been noted (Herterich et al., 2004; Lee et al., 2010), and there is a positive relationship between functionality and activities of daily living as proved by Ding et al. (2004). Likewise, isokinetic strength exercises have promoted improvements in functionality in survivors of a stroke (Loveel et al., 2009; Gerrits et al., 2009). However, there are limited studies of strength training effects on depression in survivors of a stroke.

Exercise training in general, and strength training in particular, has been promoted for leading to improvements in functionality in those affected by a stroke (Sharp et al., 1997). Physical exercises have been important for reducing the risk of mortality in cases of people who had strokes (Aidar et al., 2012), and physical activity has been shown to be beneficial for stroke cases, with the reduction in the area of ischemia (Hadidi et al., 2009).

Improvements in neuromuscular capacity have been seen in individuals with neuromuscular problems through strength training programs with both lower intensities and with high intensity exercises (Weerdensteyn et al., 2008). In this respect, none of the participants in this study had a recurrence of disease during the intervention, suggesting a tendency to decrease the risk of another stroke (Kim et al., 2001; Hadidi et al., 2009). In fact, regular exercise tends to improve, among other variables, the quality of life, the ability to work and leisure. Regular aerobic exercise also decreases the incidence of new episodes, with a consequent improvement in functional status and depression levels (Sharp and Brouwer, 1997; Carod-Artal, 2006). The studies claim that depression tends to improve with exercise, including strength training exercise (Pang et al., 2005).

In the subjects tested in this study, none had recurrence of the disease during the intervention, suggesting a tendency to decrease the risk of another accident (Hadidi et al., 2009). Evidence points towards physical activity being the main way to reduce stress in people with disabilities, in the absence of improving social and emotional aspects associated with practicing regular physical exercise (Aidar et al., 2012). These findings support improved quality of life of the subjects evaluated in this study

Moreover, there is a reduction in fatigue levels in subjects undergoing resistance training, and this reduction has a positive influence on the functionality of individuals with physical limitations due to a stroke (Van de Port et al., 2009). A resistance training program has also been shown to reduce the incidence of falls (Roth et al., 1992), as well as levels of depression in post stroke patients (Chu et al., 2004). Another study concluded that physical activities are the main way to reduce stress in people with disabilities or functional limitations, resulting in improvements in their social and emotional stability (Aidar et al., 2012). This finding corroborates the results obtained in the current study for indicators of depression. In contrast, Pang et al. (2005) demonstrated that patients with the ischemic stroke tend to perform better with weight-bearing activities. Further supporting the beneficial effects of strength training, circuit strength training has been proposed for individuals who suffered a stroke in order to maintain functionality quality of life, and to improve depression (Hadidi et al., 2009).

Traditional stroke rehabilitation programs emphasize functional training as a means to help the individual gain and maintain as much independence as possible. Training in the performance of mobility and personal care tasks, together with attempts to improve muscle strength and coordination, continue to form the central areas of focus of most rehabilitation programs. A heightened degree of physical skills required to perform these tasks, and the physiological stress placed on the reconditioned individual’s cardiovascular system while performing activities of daily living, suggest that a physiological training effect is likely to occur when these movements are performed in a sustained and systematic manner (Aidar et al., 2012). Because increased levels of physical activity are associated with a reduced risk of a stroke and cardiovascular disease, and enhanced physical and psychosocial performance, such interventions performed in a stroke rehabilitation program may have a favorable effect on the prevention of a recurrent stroke and cardiovascular events (Chu et al., 2004). All of these benefits of physical activity and strength training likely reduce the risk of depression.

## Conclusion

The results of this study indicate that improvement in strength is negatively correlated with levels of depression. Thus, strength training alone seems to reduce levels of depression in survivors of the ischemic stroke. Depression is a condition that is normally presented as a result of ischemic stroke sequelae, and is also a factor which limits the performance of activities of daily living. Strength training acts as an important means of minimizing the effects of and preventing depression caused by the ischemic stroke.

## Figures and Tables

**Table 1:** Level of deficit in the dominant or nondominant hand

Experimental Group *	**Ocurrence**
Inability light (dominant / nondominant) (%)	1 (50) / 1 (50)
Moderate disability (dominant / nondominant) (%)	2 (28.6) / 5 (71.4)
Inability high (dominant / nondominant) (%)	0 (00) / 2 (100)
Control Group *	
Inability light (dominant / nondominant) (%)	1 (50.0) / 1 (50.0)
Moderate disability (dominant / nondominant) (%)	2 (25.0) / 6 (75.0)
Inability high (dominant / nondominant) (%)	0 (00) / 3 (100)

* All subjects were right-handed

**Table 2:** Average depression rating in the pre-test and post-test using the Beck Depression Inventory

	**Pre-Test**	**Post-Test**	**p**
**Experimental Group**	17.7 ± 8.2	13.9 ± 7.4*	0,021
**Control Group**	16.9 ± 8.6	16.4 ± 7.9	0,772

* *p* ≤ *0,05 (two way ANOVA and Bonferroni Post Hoc test)*

**Table 3:** Maximum load (1RM) in kilograms (kg), after 12 weeks

	**EG pre test**	**EG pos test**	**CG pre test**	**CG pos test**
**Squat**	32.9 ± 14.2	44.4 ± 12.4*	33.9 ± 13.4	34.1 ± 13.9
**Bench Press**	19.1 ± 3.4	36.1 ± 6.4*	20.1 ± 4.2	20.3 ± 4.4
**Leg Press**	56.6 ± 16.5	80.2 ± 20.4*	54.3 ± 17.9	54.7 ± 18.3
**Military Press**	12.1 ± 4.3	21.5 ± 4.8*	13.2 ± 5.1	12.9 ± 5.3
**Crunch**	---	---	---	---
**Lat pulldowns**	11.5 ± 3.9	24.9 ± 4.1*	12.1 ± 4.5	11.9 ± 3.9
**Lunges**	29.5 ± 6.7	41.9 ± 6.7*	30.2 ± 8.2	29.7 ± 7.3

* *p*≤*0,05 (two way ANOVA and the Bonferroni Post Hoc test).*

--- Not rated

**Table 4:** Correlation between levels of depression and maximum load (1RM) in kilograms (kg)

	**“r” Spearman**
**Bar guided Squat**	− 0.727
**Machine Bech Press**	− 0.854
**Horizontal Leg Press**	− 0.901
**Military Press**	− 0.752
**Crunch**	---
**Front Lat Pulldowns**	− 0.767
**Bar guided Lunges**	− 0.889

--- Not rated
